# Systolic and Diastolic Functions After a Brief Acute Bout of Mild Exercise in Normobaric Hypoxia

**DOI:** 10.3389/fphys.2021.650696

**Published:** 2021-04-23

**Authors:** Sara Magnani, Gabriele Mulliri, Silvana Roberto, Fabio Sechi, Giovanna Ghiani, Gianmarco Sainas, Giorgio Nughedu, Romina Vargiu, Pier Paolo Bassareo, Antonio Crisafulli

**Affiliations:** ^1^Department of Medical Sciences and Public Health, University of Cagliari, Cagliari, Italy; ^2^International PhD in Innovation Sciences and Technologies, University of Cagliari, Cagliari, Italy; ^3^Department of Biomedical Sciences, University of Cagliari, Cagliari, Italy; ^4^University College of Dublin, Mater Misericordiae University Teaching Hospital, Dublin, Ireland

**Keywords:** blood pressure, cardiac pre-load, myocardial contractility, echocardiography, tissue Doppler

## Abstract

Acute hypoxia (AH) is a challenge to the homeostasis of the cardiovascular system, especially during exercise. Research in this area is scarce. We aimed to ascertain whether echocardiographic, Doppler, and tissue Doppler measures were able to detect changes in systolic and diastolic functions during the recovery after mild exercise in AH. Twelve healthy males (age 33.5 ± 4.8 years) completed a cardiopulmonary test on an electromagnetically braked cycle-ergometer to determine their maximum workload (W_max_). On separate days, participants performed randomly assigned two exercise sessions consisting in 3 min pedalling at 30% of W_max_: (1) one test was conducted in normoxia (NORMO) and (2) one in normobaric hypoxia with FiO_2_ set to 13.5% (HYPO). Hemodynamics were assessed with an echocardiographic system. The main result was that the HYPO session increased parameters related to myocardial contractility such as pre-ejection period and systolic myocardial velocity with respect to the NORMO test. Moreover, the HYPO test enhanced early transmitral filling peak velocities. No effects were detected for left ventricular volumes, as end-diastolic, end-systolic, and stroke volume were similar between the NORMO and the HYPO test. Results of the present investigation support the hypothesis that a brief, mild exercise bout in acute normobaric hypoxia does not impair systolic or diastolic functions. Rather, it appears that stroke volume is well preserved and that systolic and early diastolic functions are enhanced by exercise in hypoxia.

## Introduction

Acute hypoxia (AH) presents some challenges to the human homeostasis, especially during exercise and recovery. During AH, the human circulation experiences rapid changes in the main hemodynamic modulators (i.e., pre-load, after-load, contractility, and chronotropism), which potentially impact on cardiovascular function and regulation (Yan et al., 2007; Dedobbeleer et al., 2013; Goebel et al., 2013; Naeije and Dedobbeleer, 2013). Our group has recently shown that, among cardiovascular parameters, ventricular filling rate was one of the most sensitive to exercise in AH, as it was reduced after brief bouts of acute cycling in normobaric hypoxia (Mulliri et al., 2019, 2020). This suggested that venous return was reduced and led us to speculate that a reduction in cardiac preload took place in this setting, thereby impairing the Frank-Starling mechanism. Furthermore, this effect was not counterbalanced by any enhancement in cardiac performance.

However, one potential limit of our previous research was that we did not assess echocardiographic parameters. Instead, hemodynamics were assessed by means of impedance cardiography, which does not allow gathering data about cardiac volumes; moreover, it suffers from some limits in the analysis of systolic and diastolic functions.

In this specific area, the research is scarce. Some studies using echocardiography have provided evidence that an increase in left ventricular twist takes place in the acclimatisation at altitude. This response seems to be compensatory to maximise stroke volume (SV) when ventricular filling is impaired. Given that sub-endocardial function was maintained, the elevation in sympathetic activity was proposed as the most likely explanation for this phenomenon (Stembridge and Levine, 2019). In support to this hypothesis, findings have been recently provided that the increase in cardiac twist can be attenuated by the administration of specific β1-adrenergic antagonist, which appears to support the hypothesis that this is an appropriate response to sympathetic activation (Williams et al., 2019). Collectively, these results appear to suggest that left ventricular function is maintained or even enhanced in chronic hypoxia (Stembridge et al., 2016). Moreover, some studies conducted at rest reported an increase in ventricular twist and deformation probably due to sympathetic stimulation and/or peripheral vasodilation (Dedobbeleer et al., 2013; Goebel et al., 2013).

However, little is known about the effect of AH during exercise and recovery. Concerning diastolic functions, evidence has been provided that the diastolic trans-mitral peak early velocity gathered with Doppler is higher during exercise in hypoxia than in normoxia, so suggesting that hypoxic exercise increase ventricular diastolic function (Yan et al., 2007).

Starting from these considerations, the aim of the present study was to investigate the effect of acute dynamic exercise during normobaric hypoxia on echocardiographic parameters related left ventricular volumes, systolic, and diastolic functions. Specifically, the present investigation was devised to verify whether classical echocardiographic measures of ventricular volume confirm or reject our previous hypothesis of a reduced pre-load during the recovery from mild exercise in AH. Moreover, we aimed to verify whether Doppler and tissue Doppler measures confirmed or rejected the hypothesis that systolic and diastolic functions were affected by mild exercise conducted in acute normobaric hypoxia.

## Materials and Methods

### Participants

Twelve healthy Caucasian males aged 24–42 years [mean ± standard deviation (SD) of age 33.5 ± 4.8 years] agreed to participate in the study. All participants were physically active and were regularly involved in leisure-time sports activities such as amateur cycling and running at least 3 times/week. Their average ± SD of body mass and height were 72.5 ± 10.1 kg and 176.5 ± 3.9 cm, respectively. All were non-smokers and none of them suffered from any known diseases or were on medication at the time of the experiment. They were asked for abstaining from drinking alcohol or coffee for at least 24 h before scheduled tests. All experiments were conducted in a room at controlled temperature and humidity (22°C, relative humidity 50%).

To calculate the required sample size, we used a calculator free available on the web.^1^ The calculation was conducted using a power of 85%, an overall type 1 error of 0.05, a SD of 10%, and a 15% difference due to conditions in the studied variables. Eight subjects were needed to obtain adequate statistical power.

The study was carried out with approval from the University’s Institutional Review Board and in accordance with the Declaration of Helsinki. All the participants signed written informed consent before the beginning of the study.

### Experimental Protocol

The experimental protocol consisted in a preliminary screening test and in two experimental sessions in normoxia (test NORMO) and AH (test HYPO). Test NORMO and HYPO were randomly assigned. Randomisation was obtained using an online random sequence generator.^2^

#### Preliminary Test

All participants underwent a preliminary medical examination to assess their health status. After the medical examination, each participant underwent a cardiopulmonary exercise stress test (CPET) on an electromagnetically braked cycle-ergometer (CUSTO Med, Ottobrunn, Germany). During the CPET, oxygen uptake (V̇O_2_), carbon dioxide production (V̇CO_2_), and V_E_ were assessed with a gas analyser (Ultima CPX, MedGraphics St. Paul, MN, United States) calibrated immediately before each test accordingly to the manufacturer. Respiratory exchange ratio (RER) was also calculated as V̇CO_2_/V̇O_2_. The exercise consisted of a linear increase of workload (30 W·min^−1^), starting at 30 W, keeping a pedalling frequency of 60 rpm until exhaustion, which was considered as the point at which the subject was unable to maintain a pedalling rate of at least 50 rpm. Maximum workload (W_max_), maximum oxygen uptake (V̇O_2max_), and maximum heart rate (HR_max_) were collected. Moreover, anaerobic threshold (AT) was calculated using the *V*-slope method, which detects AT using a regression analysis of the slope of V̇CO_2_ plotted as a function of V̇O_2_ (Beaver et al., 1986). During this preliminary visit, participants familiarised with equipment and the staff of the laboratory, so allowing habituation to the environment and the ergometer that was employed in the successive experimental sessions.

### Sessions to Study Hemodynamics During Normoxia and Hypoxia

After the preliminary test (interval 3–7 days), volunteers performed randomly assigned the NORMO and the HYPO sessions pedalling on the same cycle-ergometer utilised for the CPET. NORMO and HYPO tests were separated by at least 3 days (interval 3–7 days). During both sessions, participants breathed through a mask connected to a hypoxic gas generator (Everest Summit II Generator, Hypoxico, New York, United States). This device utilises a molecular sieve system with zeolites to separate nitrogen from oxygen and allows having a gas mixture with a reduced oxygen content that can be regulated by an operator. A gas mixture with a FiO_2_ of 21% and of 13.5% (corresponding to sea level and to an altitude of about 3,500 m) was delivered during the NORMO and the HYPO, respectively. The gas mixture was constantly checked by an operator by means of oxygen analyser provided with the device (Maxtec, Handi+, Salt Lake City, UT, United States). Participants were blinded about the actual content of oxygen they were breathing. During NORMO and HYPO tests, the same cycle-ergometer used for the CPET was utilised. After wearing the mask connected to the hypoxic gas generator, participants sat on the cycle-ergometer for 3 min to collect data at rest. Then, they started pedalling for 3 min against a mild workload corresponding to the 30% of the W_max_ reached during the CPET. A recovery of 6 min was allowed after the exercise. A similar experimental approach was recently used to assess hemodynamics during metaboreflex stimulation during normobaric hypoxia (Mulliri et al., 2020).

#### Assessment of O_2_ Saturation

Peripheral blood O_2_ saturation (SO_2_) was continuously measured through finger pulse oximetry (Nonin, SenSmart X-100, Plymouth, MN, United States) to confirm that the hypoxic stimulus was effective.

#### Hemodynamic Measurement

An echocardiographic system (Vivid iq, GE Healthcare, Fairefield, CT, United States) equipped with a hand-held 3.5-MHz adult ultrasound probe was employed to assess cardiovascular functions. Heart rate (HR) was assessed as the reciprocal of the electrocardiogram R-R interval provided by the echocardiograph. Two dimensional and pulsed Doppler recording were acquired with participants in the sitting position. Measures were obtained from the apical four-chamber view. End-systolic volume (ESV) and end-diastolic volume (EDV) were calculated automatically by software using a conventional formula: 8*A*^2^/3*πL*, where *A* was the left ventricular area and *L* was the ventricular longest length (Christie et al., 1987). The ventricular area was determined by tracing along the inner edge of the endocardial targets, and the length was obtained by measuring the distance from the left ventricular apex to the midpoint of the mitral annulus. Echocardiography images were taken at rest and during the recovery from strain (i.e., at the third minute of recovery) by the same operator throughout sessions. When images were considered of good quality, a 6 s frame was recorded and then analysed offline always by the same skilled operator. For each analysis at least three beats were taken into consideration (range 3–6 beats) and data are reported as the average of the measures. Left ventricular ejection fraction (EF) was considered as: (EDV − ESV/EDV)100, and SV as: EDV − ESV.

In the same beats utilised for left ventricular volumes assessment, early and atrial transmitral filling peak velocities (Evel and Avel, respectively) and their ratio (E/A) were assessed using pulse wave Doppler (PWD) with a 5-mm PWD sample volume (3 mm) placed distal to the mitral anulus, between the mitral leaflets. The interrogation beam was aligned with mitral flow (Gardin et al., 1986; Cohen et al., 1996).

Mitral valve motion velocity during early (Em) and late (Am) diastole was determined by Doppler tissue imaging with the pulsed-wave sample volume placed at the lateral mitral anulus from the apical four chamber view. Septal early diastolic mitral anular velocities have been documented to detect impaired left ventricular diastolic functions independent of ventricular loading conditions (Nagueh et al., 1997). This technique has been already employed to analyse the effect of hypoxia on diastolic functions (Allemann et al., 2004). Moreover, systolic myocardial velocity (Sm) was determined to have a measure of longitudinal systolic function. This parameter has been found to correlate with measures of left ventricular EF and peak *dP/dt* (Correale et al., 2012). The ratio Evel/Em was used to estimate left ventricular filling pressure considering that an Evel/Em >10 is correlated with an elevated left ventricular diastolic pressure, whereas a value <8 indicates a normal pressure (Correale et al., 2012; Choudhury et al., 2017).

Aortic Doppler was also conducted from the four-chamber window to assess the pre-ejection period (PEP), which was measured as the time from the beginning of the QRS complex of the electrocardiogram and the opening of the aortic valve, and the ventricular ejection time (VET), which was assessed as the total duration of ejection period in the Doppler trace. Diastolic time (DT) was calculated by subtracting the sum of PEP and VET from the total period of the cardiac cycle (Sainas et al., 2016). We used PEP variations to have an estimate of sympathetic activity towards the left ventricle. Actually, when there is a more rapid development of intraventricular pressure, PEP shortens. Furthermore, the influence of parasympathetic activity on PEP is negligible as ventricles are not innervated by the parasympathetic nervous system. Yet, PEP is not substantially altered by changes in HR (Michael et al., 2017).

All echocardiographic calculations were done by the same expert physician, with a 5-year experience in the field. When comparing pre- and post-experiment echocardiographic measurements, and taking together all the calculation the observer did, the coefficient of variation varied from 8% (very good) to 12% (good).

A manual sphygmomanometer (Heine Gamma GP, Gilching, Germany) was placed in the non-dominant arm and systolic (SBP) and diastolic (DBP) blood pressure were measured by the same physician throughout all protocol sessions. Mean arterial blood pressure (MAP) was calculated using a formula, which takes into consideration changes in PEP, VET, and DT due to tachycardia (Sainas et al., 2016).

### Data Analysis

Data are presented as mean ± SD. The Kolmogorov-Smirnov test was employed to assess distribution normality for each variable. Since all variables were normally distributed, parametric tests were used. Paired *t* test was employed to find out differences between the NORMO and the HYPO test at rest and at recovery. Statistical analysis was performed using commercially available software (GraphPad Prism). A *p* < 0.05 was considered to determine statistical significance. For each variable, effect size (ES) was determined using Cohen’s statistic, where 0.2, 0.6, and 1.2 were interpreted as small, medium, and large effect, respectively.

## Results

Results of the CPET are reported in Table 1, while Table 2 shows the values of variables collected during the third minute of rest preceding the NORMO and the HYPO test. Statistics found out that the HYPO test induced a significant increase in Evel and in E/A ratio, whereas Avel was reduced. Moreover, DT was significantly longer during the HYPO test.

**Table 1:** Mean values ± SD of metabolic data at the anaerobic threshold (AT) and at maximum workload (W_max_) collected during the cardiopulmonary test. V̇O_2_, oxygen uptake expressed indexed for body mass (second line) as well as in absolute values (third line); V̇CO_2_, carbon dioxide production; RER, respiratory exchange ratio; VE, pulmonary ventilation; HR, heart rate. *N* = 12.

	AT	W_max_
Workload (W)	165.80 ± 31.68	244.27 ± 38.40
V̇O_2_ (ml·kg^−1^·min^−1^)	25.61 ± 3.18	38.45 ± 4.10
V̇O_2_ (ml·min^−1^)	2,008 ± 350	2,780 ± 528
V̇CO_2_ (ml·kg^−1^·min^−1^)	1,771 ± 440	3,920 ± 688
RER	1.08 ± 0.10	1.41 ± 0.05
VE (l·min^−1^)	42.75 ± 10.91	98.34 ± 18.06
HR (bpm)	146.15 ± 8.65	182.50 ± 10.50

**Table 2:** Hemodynamic values during the third minute of rest preceding the test in normoxia (NORMO) and in hypoxia with FiO_2_ at 13.5% (HYPO). *N* = 12.

	NORMO	HYPO	*p*
SO_2_ (%)	97.33 ± 1.36	98.04 ± 1.25	0.1840	
HR (bpm)	88.92 ± 14.77	80.58 ± 9.98	0.0855	
MAP (mmHg)	89.75 ± 4.55	85.83 ± 4.91	0.2106	
PEP (ms)	137.90 ± 19.27	135.50 ± 24.16	0.7176	
VET (ms)	238.02 ± 25.17	237.85 ± 38.04	0.9876	
DT (ms)	261.53 ± 55.57	347.99 ± 82.60	0.0148	
ESV (ml)	37.66 ± 12.83	39.27 ± 15.88	0.9828	
EDV (ml)	120.81 ± 17.89	120.19 ± 21.82	0.9135	
EF (%)	68.87 ± 9.14	67.85 ± 9.06	0.5927	
SV (ml)	83.13 ± 15.08	80.91 ± 13.85	0.5244	
Evel (cm·s^−1^)	54.47 ± 10.69	62.81 ± 14.54	0.0325	
Avel (cm·s^−1^)	62.05 ± 13.41	50.53 ± 7.87	0.0036	
E/A	0.91 ± 0.23	1.28 ± 0.38	0.0014	
Em (cm·s^−1^)	8.44 ± 2.36	9.14 ± 3.25	0.5695	
Am (cm·s^−1^)	8.22 ± 2.33	7.06 ± 1.46	0.1959	
Em/Am	1.09 ± 0.40	1.37 ± 0.54	0.0767	
Sm (cm·s^−1^)	10.19 ± 2.07	9.86 ± 1.52	0.6189	
Evel/Em	6.82 ± 1.60	7.48 ± 2.15	0.3926	

**Figure 1:**
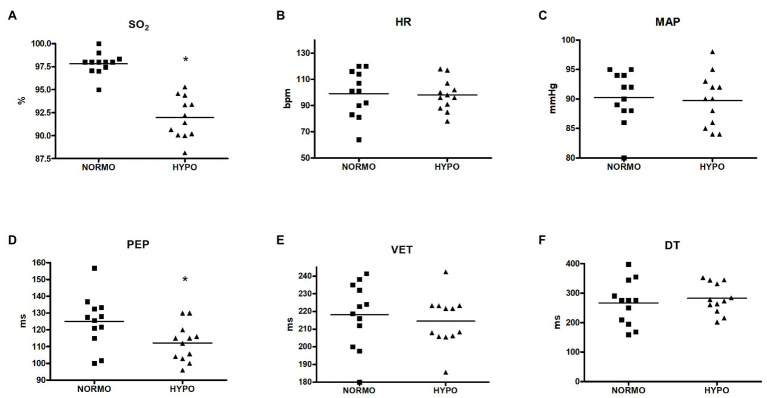
Scatter plot graphs of levels of blood O_2_ saturation (SO_2_, panel **A**), heart rate (HR, panel **B**), mean arterial pressure (MAP, panel **C**), pre-ejection period (PEP, panel **D**), ventricular ejection time (VET, panel **E**), and diastolic time (DT, panel **F**) during the recovery from sessions of exercise in normoxia (NORMO) and in normobaric hypoxia with a FiO_2_ of 13.5% (HYPO). *N* = 12. *p < 0.05 vs. NORMO test.

Figure 1 exhibits values of variables collected during recovery from the HYPO and the NORMO test. Panel A shows that SO_2_ was significantly reduced during the HYPO test (97.81 ± 1.20 vs. 91.97 ± 2.23% for the NORMO and the HYPO test, respectively, *p* < 0.001, ES = 0.85), while HR (99.08 ± 17.58 vs. 98.00 ± 12.01 bpm, *p* = 0.8628, ES = 0.01) and MAP (90.25 ± 4.41 vs. 89.75 ± 4.49 mmHg, *p* = 0.5412, ES = 0.02) were unaffected by conditions (panels B and C, respectively). Panel D demonstrates that PEP was shorter during the HYPO test than during the NORMO test (124.96 ± 15.30 vs. 112.21 ± 11.00 ms for the NORMO and the HYPO test, respectively, *p* = 0.0178, ES = 0.24), whereas VET (218.14 ± 18.41 vs. 214.60 ± 14.33 ms, *p* = 0.6040, ES = 0.05) and DT (266.40 ± 74.97 vs. 282.59 ± 51.08 ms, *p* = 0.4321, ES = 0.06) were not influenced by condition (panels E and F).

**Figure 2:**
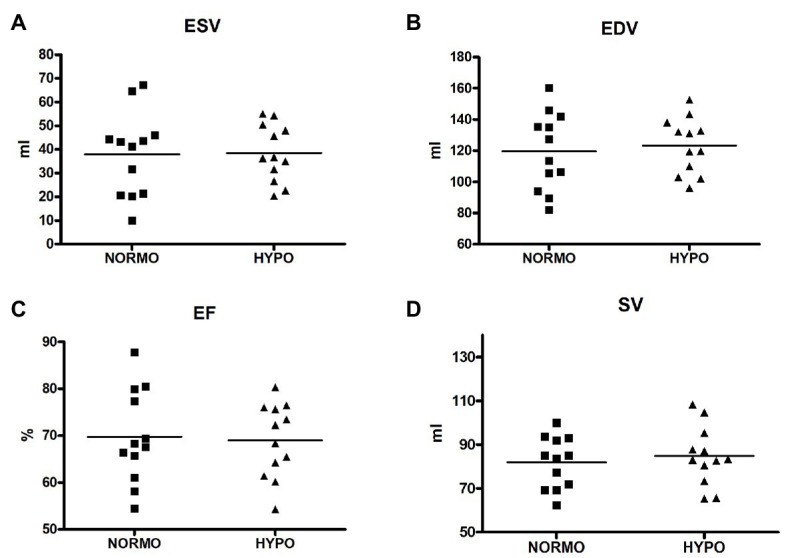
Scatter plot graphs of levels of end-systolic volume (ESV, panel **A**), end-diastolic volume (EDV, panel **B**), ejection fraction (EF, panel **C**), and stroke volume (SV, panel **D**) during the recovery from sessions of exercise in normoxia (NORMO) and in normobaric hypoxia with a FiO_2_ of 13.5% (HYPO). *N* = 12.

Figure 2 illustrates that ESV, EDV, EF, and SV were not significantly different between conditions (panels A, B, C, and D, respectively). In detail, ESV was 37.88 ± 17.73 vs. 38.56 ± 12.04 ml for the NORMO and the HYPO test, respectively (*p* = 0.7993, ES = 0.01), EDV was 119.77 ± 24.81 vs. 123.30 ± 17.88 ml (*p* = 0.3943, ES = 0.04), EF was 69.70 ± 9.93 vs. 68.99 ± 7.94% (*p* = 0.6219, ES = 0.01), and SV was 81.88 ± 117.79 vs. 84.73 ± 13.40 ml (*p* = 0.2312, ES = 0.05).

**Figure 3:**
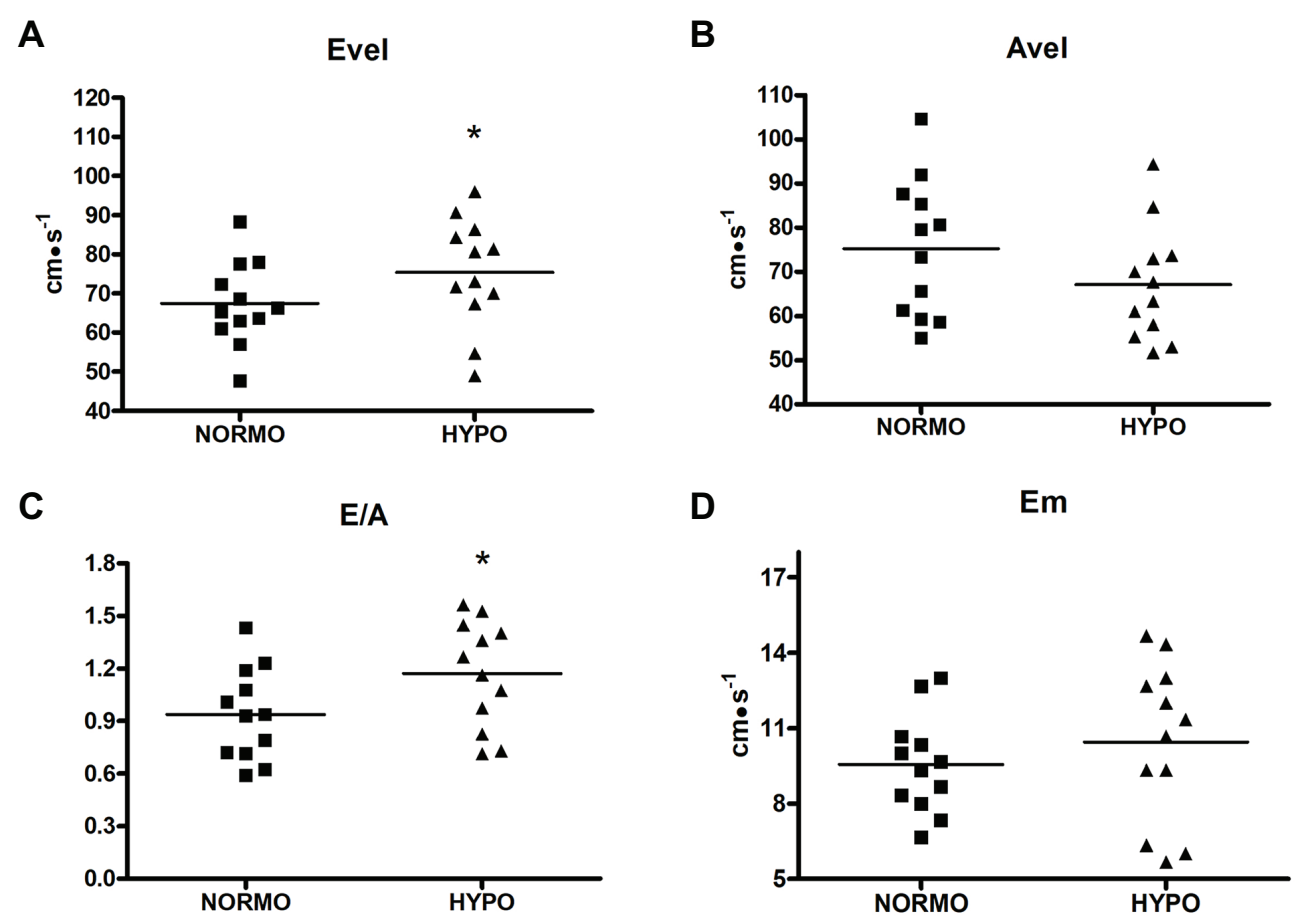
Scatter plot graphs of levels of early transmitral filling peak velocity (Evel, panel **A**), late transmitral filling peak velocity (Avel, panel **B**), their ratio (E/A, panel **C**), and early diastolic mitral valve motion velocity (Em, panel **D**) during the recovery from sessions of exercise in normoxia (NORMO) and in normobaric hypoxia with a FiO_2_ of 13.5% (HYPO). *N* = 12. *p < 0.05 vs. NORMO test.

HYPO test increased Evel with respect to the NORMO test (75.41 ± 14.01 vs. 67.41 ± 10.69 cm·s^−1^, *p* = 0.0478, ES = 0.16. Figure 3, panel A). Similarly, E/A was also increased by the HYPO with respect to the NORMO test (1.17 ± 0.30 vs. 0.93 ± 0.26, *p* = 0.0315, ES = 0.20, Figure 3, panel C), while Avel and Em (panels B and D, respectively) were not different between conditions (75.27 ± 15.60 vs. 67.13 ± 12.95 cm·s^−1^, *p* = 0.1603, ES = 0.14, and 9.55 ± 1.94 vs. 10.44 ± 3.15 cm·s^−1^, *p* = 0.3343, ES = 0.08 for Avel and Em during the NORMO and the HYPO test, respectively).

**Figure 4:**
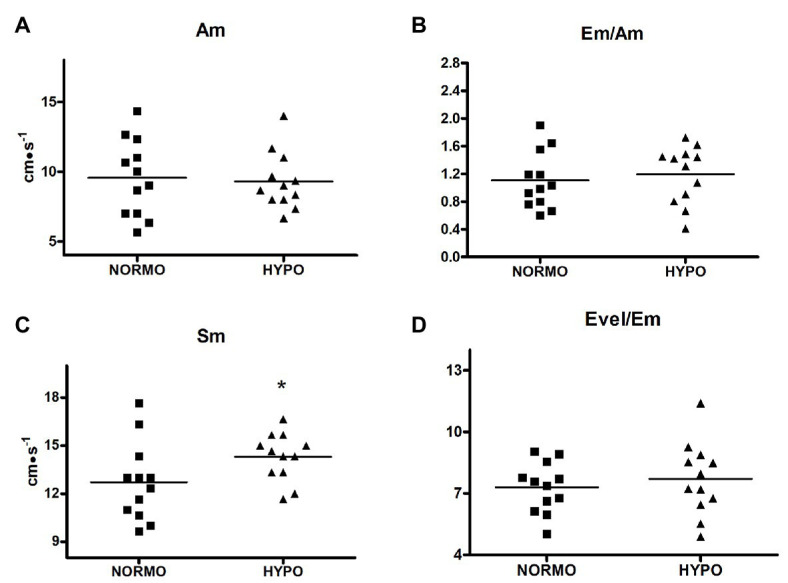
Scatter plot graphs of levels of late diastolic mitral valve motion velocity (Am, panel **A**), ratio between early and late diastolic mitral valve motion velocities (Em/Am, panel **B**), systolic myocardial velocity (Sm, panel **C**), and ratio between early transmitral filling peak and early mitral valve diastolic velocities (Evel/Em, panel **D**) during the recovery from sessions of exercise in normoxia (NORMO) and in normobaric hypoxia with a FiO_2_ of 13.5% (HYPO). *N* = 12. *p < 0.05 vs. NORMO test.

Finally, Figure 4 shows that Sm (panel C) was higher during the HYPO than during the NORMO test (14.30 ± 1.49 vs. 12.72 ± 2.445 cm·s^−1^, *p* = 0.0431, ES = 0.20), whereas Am (panel A), Em/Am (panel B), and Evel/Em were not different between conditions (9.55 ± 2.75 vs. 9.30 ± 2.05 cm·s^−1^, *p* = 0.7387, ES = 0.02; 1.10 ± 0.40 vs. 1.19 ± 0.41, *p* = 0.4623, ES = 0.05; and 7.29 ± 1.23 vs. 7.71 ± 1.77, *p* = 0.4875, ES = 0.07 for Am, Em/Am, and Evel/Em during the NORMO and the HYPO test, respectively).

## Discussion

In the present study, a group of healthy physically active male subjects performed a brief, mild exercise bout during acute hypoxia to study the cardiovascular response during the following recovery. The fact that our experimental approach was capable of effectively inducing hypoxemia was testified by SO_2_, which substantially dropped during the HYPO session, as shown by Figure 1 (panel A).

The main purpose of the present investigation was to verify whether classical echocardiographic measures of ventricular volume confirmed or rejected our previous hypothesis of a reduced pre-load during the recovery from mild efforts conducted in AH. Another aim was to verify whether Doppler and tissue Doppler measures confirmed or rejected the hypothesis that, during the recovery from exercise in AH, systolic and diastolic functions increased due to sympathetic activation.

Based on results, we must reject the first hypothesis. Indeed, EDV was similar between conditions, thereby indicating that there were not reductions in cardiac pre-load in participants. Moreover, ESV, EF, and SV were not different between the NORMO and the HYPO tests. These findings suggest that a brief bout of mild exercise in AH could not cause any modification in cardiac variables related to heart volumes.

This finding appears to contradict our recent investigation, where, in healthy humans, a reduction in the capacity to increase SV due to impairment in ventricular filling rate was found in response to the muscle metaboreflex activation after exercise in AH (Mulliri et al., 2020). In this paper, it was hypothesised that the reduced ventricular filling rate was the consequence of an increase in the production of metabolite-mediated venodilation, such as NO, adenosine, and prostaglandin derived factors, which exerted vasodilatory activity in the venous bed (Marshall, 2015; Dinenno, 2016) and prevented the recruitment of the Frank-Starling mechanism. It should however be considered that, in the quoted study, hemodynamics were studied during the metaboreflex stimulation, which causes a substantial sympathetic activation. Thus, the present and the former studies are quite different in the experimental approach, and their results can be only partially comparable. Moreover, in our previous investigation, we could not conduct any echocardiography assessment. Thus, out hypothesis was speculative. Further study using echocardiography, during the metaboreflex, should be conducted in order to verify whether metaboreflex-induced sympathetic activation after exercise in AH reveals any impairment in EDV and in the capacity to vasoconstrict the venous bed in healthy humans.

Another result of the present research was that, during the recovery of the HYPO test, PEP significantly shortened in comparison with the NORMO test (Figure 1, panel D). This indicated that, in this setting, there was an increase in myocardial contractility, as this parameter is inversely related to the development of intraventricular pressure. Concerning the influence of autonomic activity, it is to be highlighted that PEP responds only to sympathetic stimulation, since the influence of parasympathetic tone is negligible on ventricles. Moreover, PEP does not depend on changes in HR (Michael et al., 2017). Thus, the PEP shortening could be the consequence of an increase in sympathetic tone, although other phenomena may have taken part in the myocardial contractility enhancement (see the following part of Discussion).

The fact that after the exercise bout in AH an increase in contractility took place is also confirmed by the Sm velocity gathered by tissue Doppler, which was faster during the HYPO as compared to the NORMO test (Figure 4, panel C). Sm velocity at the lateral mitral anulus is correlated with ventricular peak *dP/dt* and it can be considered an index of inotropism (Correale et al., 2012). Actually, ventricular systole pulls down the atrio-ventricular plane, and it seems reasonable to assume that the displacement of this plane is an expression of the myocardial contractility (Höglund et al., 1988). In support to the notion that Sm is related to myocardial performance there are findings that mitral anulus systolic excursion is reduced in patients with ventricular dysfunction (Grue et al., 2018; Berg et al., 2020).

It is to be noticed that, during the rest period of the HYPO session, neither PEP nor Sm was affected by the administration of the hypoxic gas mixture (see Table 2). This result suggests that, rather than hypoxia *per se*, the exercise bout in AH was the real responsible for changes in both parameters related to myocardial contractility. It is also possible that sympathetic activation was not the only responsible for the enhanced myocardial contractility. The concept that several substances produced during exercise in AH can enhance inotropism independently from sympathetic activity has been the subject of active research in the last years. Specifically, apart from sympathetic tone, during AH some metabolic products, such as apelin, may exert positive inotropic effect (Calbet et al., 2009), thus explaining why we noticed an increase in Sm only after exercise in AH and not at rest. For instance, recent findings demonstrated that left ventricular twist mechanic is not impaired by acute hypoxia and that endocardial dysfunction did not occur during AH (Williams et al., 2019). Moreover, it should be mentioned that during exercise in ischemic conditions several metabolites are produced, and these metabolites can trigger the phenomenon termed *ischemic preconditioning*, which confers cardioprotection and favourable hemodynamics effects (i.e., increase in myocardial performance and vasodilation) within few minutes (Marongiu and Crisafulli, 2014). It is then conceivable to hypothesise that exercise in AH leads to a similar metabolites production as during ischemia. To the best of our knowledge, this possibility has never investigated before and it may represent an intriguing field of research in a physiological and clinical perspective.

Whatever the cause responsible for the increased myocardial performance, results of the present investigation confirm that contractile function is preserved after mild exercise bouts in AH and that SV is well preserved by mechanisms, which are only partially known. While a decrease in SV, during acclimatisation at high altitude has been several times reported, this phenomenon is usually not observed during AH (Stembridge et al., 2016).

Another result of the present investigation was that diastolic function was significantly modified by AH both at rest and after the exercise bout. Regarding results at rest, it appears that the hypoxic gas administration shifted ventricular filling from the late to the early phase. This can be at least partially explained by the longer DT during the HYPO in comparison with the NORMO test. This was the result of the slight reduction in HR occurring during the rest period of the HYPO test, which, although insignificant with respect to the NORMO test, nonetheless led to a longer cardiac cycle with respect to the NORMO test. Considering that PEP and VET were quite similar between conditions, then it followed that DT was longer in the HYPO test. We cannot however rule out that the hypoxic condition could improve early diastolic function by any unknown mechanism able to enhance ventricular relaxation. To the best of our knowledge, there are no studies focusing on the potential effect of AH on the myocardial early diastolic proprieties, and further research is warranted in this area. It should however be acknowledged that the presence of any hypoxic-mediated mechanism was unlikely in our setting at rest as the hypoxic stimulus did not significantly reduce SO_2_, so indicating that the hypoxic stress was mild.

A different diastolic behaviour between tests was present also during the recovery phase. Indeed, Evel and E/A were significantly higher after AH. An increase in Evel, during exercise in AH, has been already reported in the scientific literature (Yan et al., 2007), but the phenomenon has been never replied by other groups to date. Authors of the quoted paper suggested that acute hypoxic exercise increased diastolic function, although no explanation for the phenomenon was provided. Our results seem to confirm these previous findings. Moreover, our results suggest that the increased contractility could be at least in part responsible for it. Both PEP and Sm indicated that, during the HYPO test, myocardial contractility was more elevated with respect to the NORMO test. It was observed that the energy generated during systole is stored in the extracellular collagen matrix and then released during diastole, thereby supporting ventricular filling (Notomi et al., 2008). Furthermore, it was also proposed that the atrio-ventricular plane acts like a piston driven by ventricular contraction and that its movement during systole pulls blood from the venous tree to the atria (Arutunyan, 2015). In short, when the ventricles contract, the A-V plane descends towards the apex, while the pulmonary veins remain fixed in the mediastinum. The descent of the A-V plane aspirates blood from the pulmonary circulation and generates one of the forces able to fill the atria (Chung et al., 2015). In humans, it has been demonstrated that up to 70% of atrial filling occurs during ventricular emptying and is driven by ventricular longitudinal contraction (Steding-Ehrenborg et al., 2013).

Then, the increased myocardial performance during the HYPO test may have enhanced early diastolic filling with at least two different phenomena: (a) an increase in the energy generated during systole and recoiled during diastole and (b) a more efficient A-V displacement, which allowed a more effective atrial filling.

A third phenomenon that could theoretically affect diastolic filling could be the increase in left ventricular filling pressure due to hypoxia-induced pulmonary vasoconstriction (Naeije and Dedobbeleer, 2013; Stembridge et al., 2016). We employed Evel/Em to estimate the left ventricular filling pressure, but we did not find out any significant difference between the HYPO and the NORMO test. It can be then concluded that a brief bout of exercise in AH cannot significantly affect left ventricular filling pressure.

### Limitations of the Study

Some limitations of the present investigation should be honestly acknowledged.

In detail, echocardiographic measures were conducted only during recovery and not during exercise. This is because, in a pilot study, we could not collect good images during cycling mainly because of chest movements due to respiration. Probably, the best position in this kind of research is the recumbent one. However, this position is not very natural as normally individuals exercise standing or sitting, as in the present investigation. Moreover, the recumbent position increases venous return, thereby affecting EDV and diastolic functions.

It should be pointed out that diastolic measures obtained with tissue Doppler yielded different results with respect to trans-mitral Doppler. Specifically, while Evel was significantly increased by the HYPO test, Em was not affected by this condition. One explanation for this different outcome could be that tissue Doppler measures are highly dependent on the angle between scan beam and the vector of ventricular motion, which should be parallel (Bassareo et al., 2010). It is then possible that chest movements due respiration after effort may have rendered problematic tissue Doppler measures in our experimental setting, thus affecting assessment precision.

Another limit could be that we did not directly assess myocardial inotropism. Instead, indirect measures were used, i.e., PEP and Sm. However, the direct assessment of myocardial inotropism is problematic in humans as it requires the use of invasive technologies, which are not advisable in study such the present one.

Finally, the present study was conducted in healthy male individuals, thus its results cannot be applicable for elderly people, for females, or for patients suffering from any disease. Further research in different groups of individuals is needed to have a clearer picture of the hemodynamic consequences of hypoxia during exercise in these sub-groups.

## Conclusion

Overall, the results of the present investigation support the hypothesis that a brief exercise bout of mild intensity in acute normobaric hypoxia does not impair systolic or diastolic functions. Rather, it appears that SV is well preserved, thanks to an improvement in inotropism and in early diastolic function. It remains to be ascertained whether the described improvement in diastolic function is a direct consequence of the enhanced systolic activity or it is due to an unknown metabolic process triggered by exercise in hypoxia. Taking into consideration that exercise in hypoxia has been proposed as a useful tool for training as well as for therapeutic purposes, its effects should be further investigated to better understand its hemodynamics and its capacity to product regulating metabolites.

## Data Availability Statement

The raw data supporting the conclusions of this article will be made available by the authors, without undue reservation.

## Ethics Statement

Ethical review and approval was not required for the study on human participants in accordance with the local legislation and institutional requirements. The patients/participants provided their written informed consent to participate in this study.

## Author Contributions

AC, SM, GM, and PB conceived the protocol, conducted experiments, analysed data, ran statistics, interpreted results, and wrote the manuscript. SR, FS, GS, GG, GN, and RV conducted experiments, analysed data, ran statistics, and interpreted results. All authors contributed substantially to the article and approved its final form.

### Conflict of Interest

The authors declare that the research was conducted in the absence of any commercial or financial relationships that could be construed as a potential conflict of interest.

## Footnotes

^1^https://clincalc.com/stats/samplesize.aspx

^2^https://www.random.org/sequences/
